# Association between vitamin D deficiency and adrenal gland, kidney function, indicators related to cardiovascular function in hypertensive patients

**DOI:** 10.3389/fnut.2026.1741253

**Published:** 2026-06-03

**Authors:** Minglei Zhang, Qunyuan Wang, Cuiping Pan, Ruizhe Li, Yining Liu, Yufeng Jin, Zongbin Li

**Affiliations:** 1 Senior Department of Cardiology, The Sixth Medical Center of Chinese PLA General Hospital, Beijing, China; 2 School of Medicine, Nankai University, Tianjin, China; 3 The Fifth Clinical College, Anhui Medical University, Hefei, China; 4 Chinese PLA Medical College, Beijing, China

**Keywords:** adrenal gland, cardiovascular function, hypertensive patients, kidney function, vitamin D deficiency

## Abstract

**Background:**

Previous research has established an association between vitamin D deficiency and various non-skeletal metabolic disorders. However, the potential association between vitamin D status and specific clinical manifestations including renal dysfunction, primary aldosteronism (PA), and cardiovascular function-related indicators in patients with hypertension remains poorly elucidated.

**Methods:**

A total of 250 consecutive patients with hypertension who were hospitalized in the Senior Cardiology Department of the Sixth Medical Center of the Chinese PLA General Hospital were enrolled in this study. Volunteers currently supplementing with vitamin D have been excluded. Fasting peripheral venous blood samples were collected from all participants for routine biochemical assays. All participants underwent a comprehensive set of examinations, including transthoracic echocardiography, ambulatory blood pressure monitoring, and routine urinalysis. Serum levels of 25-hydroxyvitamin D (25-OH-D) were measured to evaluate vitamin D status. Relevant clinical and physiological indicators were recorded. Differences in auxiliary examination results and physiological indicators between subgroups were compared and analyzed. The correlations of serum 25-OH-D levels with renal function parameters, cardiovascular function-related indicators, and PA status were further investigated.

**Results:**

Of all participants, 165 patients had vitamin D deficiency (serum 25-OH-D < 50 nmol/L), whereas 85 patients had non-deficient vitamin D status (serum 25-OH-D ≥ 50 nmol/L). There were no statistically significant differences in age, gender, and body mass index (BMI) between the two groups. Compared with the non-deficient group, patients in the vitamin D deficiency group (25-OH-D < 50 nmol/L) had significantly lower levels of hematocrit, red blood cell count, hemoglobin, serum calcium, urinary uric acid, and urinary creatinine, as well as significantly higher levels of serum sodium, supine aldosterone concentration, supine aldosterone-to-renin ratio (ARR), and upright aldosterone concentration (all *p* < 0.05). Binary logistic regression analysis revealed that PA was significantly associated with vitamin D deficiency (*OR* 0.210, 95% *CI* 0.045–0.974, *p* = 0.046). Multivariate linear regression analysis demonstrated that serum 25-OH-D levels were independently and inversely associated with estimated glomerular filtration rate (eGFR) (*β* = −4.348, *t* = −3.082, *p* = 0.002). Patients with chronic kidney disease (CKD) had a significantly larger left atrial anteroposterior diameter [39(36,42) vs. 36(32,39) mm, *p* = 0.001] and pulmonary artery diameter [24(22,26) vs. 23(21,25) mm, *p* = 0.010], along with a higher 24-h mean systolic blood pressure (131 ± 11 vs. 127 ± 11 mmHg, *p* = 0.042), but a lower 24-h mean diastolic blood pressure (73 ± 10 vs. 77 ± 9 mmHg, *p* = 0.042), compared with those without renal dysfunction.

**Conclusion:**

After adjustment for traditional cardiovascular risk factors, PA remained independently associated with vitamin D deficiency in patients with hypertension. Vitamin D and retinol-binding protein were significantly associated with renal dysfunction in this patient population.

## Introduction

Vitamin D deficiency is a prevalent nutritional issue affecting approximately half of the global population (1–3). This deficiency is not only associated with skeletal metabolic disorders but also linked to a wide range of non-skeletal metabolic diseases. Vitamin D is a fat-soluble vitamin and a steroid derivative. Natural vitamin D exists in two primary forms: D_2_ and D_3_. Adequate ultraviolet-B (UVB, 290–315 nm) exposure enables cutaneous synthesis of vitamin D₃ from 7-dehydrocholesterol-a cholesterol derivative stored in the epidermis and regarded as the vitamin D_3_ precursor. Vitamin D is primarily acquired through dietary intake and sunlight exposure, with adequate sunlight being sufficient to meet the body’s requirements (4). Vitamin D_2_ is found in plants, fungi, yeast, and invertebrates, whereas vitamin D_3_ is abundant in vertebrate sources such as fish liver oil, egg yolk, liver, and kidney tissues. These dietary sources supply essential nutrients that are critical for the maintenance of systemic health. The main form of vitamin D in plasma is 25-hydroxyvitamin D (25-OH-D). Although other tissues and cells express the extrarenal 1-hydroxylase, renal CYP27B1 activity primarily determines circulating 1,25-(OH)_2_-D levels (5). Virtually the biological actions of vitamin D are considered to be mediated by its active metabolite 1,25-(OH)_2_-D. However, because of its short half-life, 25-OH-D is considered as the standard biomarker for assessing the vitamin D status. Optimal levels are considered to be above 75 nmol/L (30 ng/mL), while insufficiency is indicated by levels between 52.5 and 72.5 nmol/L (21–29 ng/mL). The deficiency is defined when levels are below 50 nmol/L (20 ng/mL), and severe deficiency is defined when levels are below 25 nmol/L (10 ng/mL) (6, 7). Primary aldosteronism (PA) is characterized by a high prevalence of 25-OH-D deficiency, which may be partially attributed to aldosterone-induced secretion of parathyroid hormone (PTH), increasing renal catabolism of 25-OH-D, and vitamin D play a direct inhibitory effect on aldosterone synthase expression (8–10). 25-OH-D deficiency/insufficiency is highly prevalent across all stages of chronic kidney disease (CKD) and is also common in patients with cardiovascular disorders. Mechanistically, 1,25-(OH)_2_-D negatively regulates the renin-angiotensin-aldosterone system (RAAS), and vitamin D deficiency abrogates this inhibitory effect, resulting in renin overexpression and RAAS hyperactivation, which in turn contributes to the development of hypertension and left ventricular hypertrophy (1, 2, 11, 12). Hypertension is, at least in part, attributable to persistent RAAS activation, which induces endothelial dysfunction and accelerates the progression of arteriosclerosis. Vitamin D deficiency exacerbates this process via increased oxidative stress and impaired antioxidant capacity, impairing endothelium-dependent vasodilation, mediated in part through reduced endothelial nitric-oxide synthase (eNOS) expression and diminished nitric oxide (NO) bioavailability (13, 14). In addition, a positive correlation exists between vitamin D deficiency and the incidence of atrial fibrillation. Vitamin D–vitamin D receptor (VDR) signaling in cardiomyocytes exerts antioxidant effects that attenuate atrial reactive oxygen species (ROS) production. Conversely, vitamin D deficiency abolishes this antioxidant protection, facilitating pro-inflammatory and pro-arrhythmic substrate formation (15). Vitamin D can directly or indirectly modulate systemic inflammation, and indirectly inhibit atherogenesis.

In the vascular endothelium, 1,25-(OH)_2_-D downregulates endothelial cyclooxygenase-1 (COX-1), which reduces the release of endothelium-derived contracting factors and thereby regulates vascular tone (5). Previous studies have demonstrated that 25-OH-D deficiency is an independent predictor of mortality in patients with chronic heart failure, and interventional studies have shown that vitamin D supplementation improves prognosis in these patients (16). However, randomized controlled trials have yet to provide conclusive evidence that vitamin D supplementation prevents the onset of cardiovascular disease (2).

With advances in diagnostic technology, particularly following the adoption of the plasma aldosterone-to-renin ratio (ARR) for screening, the prevalence of PA among hypertensive populations has increased from the previously reported <1% to at least 5% in current cohorts, and may even reach as high as 13% (1, 6, 7, 17–19). PA is now recognized as the most common endocrine cause of secondary hypertension, rather than a rare disease entity. In light of these findings, we aimed to investigate the correlation between circulating vitamin D concentrations and renal dysfunction, primary aldosteronism, hypertension, and cardiac dysfunction. Understanding this relationship is crucial for developing effective strategies to address vitamin D deficiency and its associated adverse clinical outcomes.

## Materials and methods

### Research object

#### Ethical statement

This study recruited volunteers through open continuous recruitment, which was approved by the Ethics Committee of Chines PLA General Hospital (approval number HZKY-PJ-2021-5). Written informed consent was obtained from all volunteers after a comprehensive explanation of the study’s objectives, procedures, potential risks, and benefits.

#### General information

Participants were consecutively recruited at the Sixth Medical Center, Chinese People’s Liberation Army General Hospital, between April 2021 and April 2022. Volunteers currently supplementing with vitamin D have been excluded. After screening, 250 volunteers (147 males, 58.8%; 103 females, 41.2%) were enrolled (Table 1).

**Table 1:** Sex frequency distribution of the 250 enrolled volunteers.

Gender	Frequency	Proportion (%)	Cumulative frequency	Cumulative proportion (%)
Male	147	58.80	147	58.80
Female	103	41.20	250	100.00
Total	250	100.00	–	–

##### Inclusion criteria

(1) Systolic/diastolic blood pressure ≥140/90 mmHg or established diagnosis of hypertension.
(2) Adequate literacy, writing, comprehension and communication skills.
(3) Fully understand the research content and signed a written informed consent provided.

##### Exclusion criteria

(1) History of respiratory failure, cardiac arrest, severe respiratory disease, decompensated hepatic disease (including cirrhosis), or end-stage renal disease requiring maintenance dialysis.
(2) Active infection or other acute inflammatory condition within 4 weeks.
(3) Concurrent participation in another clinical trial.

### Research methodology

#### Data collection

Upon enrolment, all participants underwent a standardized baseline evaluation. Demographic and clinical data such as age, body mass index (BMI), systolic and diastolic blood pressure (SBP/DBP), and heart rate (HR) were recorded. Urinalysis, 24-h ambulatory blood pressure monitoring (ABPM), and transthoracic echocardiography were performed for all participants. ABPM was performed with 30-min intervals over a 24-h period; derived parameters included 24-h, daytime, and nighttime mean SBP/DBP and HR. Echocardiographic measurements included left ventricular ejection fraction, stroke volume, average HR, pulmonary artery diameter, interventricular septal thickness, and aortic flow velocity.

After an overnight fast of more than 12 h, peripheral venous blood samples was collected for a comprehensive laboratory testing, including complete blood count (white blood cell count, red blood cell count, and platelet counts, hemoglobin level, among other indices), liver and renal function assays, serum electrolytes, fasting lipids profiles, cardiac troponins, N-terminal pro-B-type natriuretic peptide (NT-proBNP), and serum 25-hydroxyvitamin D (25-OH-D) concentrations, serum 25-OH-D levels were used to assess participants’ vitamin D status (17).

#### Definition and diagnostic work-up of primary aldosteronism (PA)

Primary aldosteronism (PA) is an adrenal cortical disorder characterized by autonomous aldosterone overproduction, which leads to sodium retention, potassium wasting, expanded extracellular fluid volume, suppressed renin-angiotensin system (RAS) activity, and a clinical phenotype of hypertension (with or without hypokalaemia).

##### Screening conditions

(1) Office blood pressure ≥140/90 mmHg despite ≥ 2 maximally-tolerated antihypertensive agents used in adequate doses for over 12 weeks.
(2) Contrast-enhanced CT or MRI of the adrenals showing either a unilateral aldosterone-producing adenoma or bilateral adrenal hyperplasia that is biochemically correlated with autonomous aldosterone secretion.

Patients who meet any of the above conditions, especially the second condition, should undergo screening tests.

##### Blood sampling conditions

(1) Two weeks before screening, it is recommended that patients avoid excessive intake of salt and bad habits such as chewing tobacco. Blood samples were taken at 6–10 a.m. After awakening, the patient remains upright position (sitting, standing or walking) for ≥2 h, then sits quietly for 5–15 min before blood collection.
(2) Hemolysis must be avoided.
(3) Samples are transported at room temperature (placing tubes on ice converts inactive to active renin).

##### Screening procedures

Patients with aldosterone/renin ratio (ARR) ≥ 3.7 and plasma aldosterone concentration (PAC) ≥ 15 ng/dL should undergo further confirmation. Calculation of ARR is to divide the plasma aldosterone concentration (PAC) by direct renin concentration (DRC) (20).

##### Confirmation of PA

Confirmation of PA was performed using the captopril challenge test (CCT).

Fifty milligrams oral captopril; plasma aldosterone and direct renin concentration are measured at baseline and 1 and 2 h post-dose. The diagnoses of PA was assessed using 4 CCT criteria post-CCT: (1) direct renin concentration <1 nIU/mL and aldosterone suppression is absent (<30% reduction); (2) ARR > 20; (3) direct renin concentration <1 nIU/mL; (4) PAC still >11 ng/dL.

#### Indicator evaluation and analysis

Primary continuous outcome measure: estimated glomerular filtration rate (eGFR) calculated using the Modification of Diet in Renal Disease (MDRD) equation. The correlation between serum 25-OH-D levels and eGFR was assessed using multiple linear regression analysis.

Primary categorical outcome measure: renal insufficiency, defined as eGFR <60 mL/(min·1.73m^2^), Participants with an eGFR ≥60 mL/(min·1.73m^2^) served as the referent (no renal insufficiency).

#### Statistical methods

Statistical analyses were performed using SPSS version 25.0 (IBM SPSS Statistics, Armonk, NY, United States). Quantitative variables were tested for normality via the Shapiro–Wilk test and for homogeneity of variance via the Levene test. Normally distributed data are presented as mean ± standard deviation (SD), non-normally distributed data as median and quartiles [ $M(P_{25},P_{75})$ ], and categorical data are presented as *n* (%).

Between-group comparisons of continuous variables were performed using the independent samples *t*-test (for normally distributed data) or nonparametric tests (for non-normally distributed data). For categorical variables, comparisons were made using the chi-square test or Fisher’s exact probability test, as appropriate. The categorical primary outcome measure was analyzed using binary logistic regression analysis, and the continuous primary outcome measure was analyzed using multiple linear regression analysis. A two-sided *p*-value <0.05 was considered statistically significant.

## Results

### Baseline result analysis

Among the 250 volunteers enrolled, vitamin D status was predominantly characterized by deficiency and insufficiency. Vitamin D deficiency was the most prevalent category, affecting 130 individuals (52.0% of the cohort) (Table 2).

**Table 2:** Frequency distribution of vitamin D levels in 250 volunteers.

25-OH-D (nmol/L)	Frequency	Percentage	Cumulative frequency	Cumulative percentage
25-OH-D < 25	35	14.0	35	14.0
25 ≤ 25-OH-D < 50	130	52.0	165	66.0
50 ≤ 25-OH-D < 75	63	25.2	228	91.2
25-OH-D ≥ 75	22	8.8	250	100.0
Total	250	100.0	–	–

Volunteers with 25-OH-D levels <50 nmol/L exhibited significantly lower levels of hematocrit [0.40(0.37,0.44) vs. 0.42(0.40,0.45), *p* = 0.007], red blood cell count (4.51 ± 1.46 vs. 4.69 ± 1.30, *p* = 0.038), hemoglobin [133(122,149) vs. 144(133,155), *P* < 0.001], serum calcium [2.28(2.21,2.35) vs. 2.31(2.26,2.37), *p* = 0.020], uric acid [3.35(2.28,5.01) vs. 4.04(2.96,5.57), *p* = 0.018], and urinary creatinine [10.3(7.7,12.8) vs. 11.4(9.2,15.4), *p* = 0.004], compared to those with 25-OH-D ≥ 50 nmol/L. In contrast, serum sodium levels were significantly higher in the 25-OH-D < 50 nmol/L group [140.7(139.3,142.2) vs. 140.2(138.2,141.2), *p* = 0.004]. In the supine position, participants with 25-OH-D < 50 nmol/L had significantly higher aldosterone levels [9.42(6.49,14.40) vs. 7.92(5.72,19.89) *p* = 0.006], aldosterone-to-renin ratio (ARR) [1.63(0.55,4.80) vs. 1.01(0.50,2.81), *p* = 0.049], and upright aldosterone levels [13.91(9.21,20.20) vs. 11.70(7.26,17.01), *p* = 0.018], compared to the 25-OH-D ≥ 50 nmol/L group. The inter-group difference was statistically significant. No statistically significant differences were observed between the two groups in 24-h average systolic blood pressure, 24-h average diastolic blood pressure, 24-h average heart rate, or left ventricular ejection fraction (Table 3).

**Table 3:** Baseline characteristics of volunteers.

Characteristics	25-OH-D < 50 nmol/L (*n* = 165)	25-OH-D ≥ 50 nmol/L (*n* = 85)	*P*-value
Baseline data			
Age [ $M(P_{25},P_{75}),\text{years}$ ]	58 ± 16	57 ± 15	0.539
Male sex, *n* (%)	90(54.55)	57(67.06)	0.057
BMI ( $\bar{x}\pm s,\mathrm{kg}/m^{2}$ )	25.85 ± 3.18	26.38 ± 3.49	0.234
Complete blood count			
Neutral lymphatic ratio (NLR) [ $M(P_{25},P_{75})$ ]	1.94(1.43,2.55)	1.87(1.51,2.63)	0.967
Red blood cell count ( $\bar{x}\pm s,10^{12}/L$ )	4.51 ± 1.46	4.69 ± 1.30	0.038
Hemoglobin [ $M(P_{25},P_{75}),g/L$ ]	133(122,149)	144(133,155)	<0.001
Hematocrit [ $M(P_{25},P_{75}),L/L$ ]	0.40(0.37,0.44)	0.42(0.40,0.45)	0.007
Platelet count ( $\bar{x}\pm s,10^{9}/L$ )	223 ± 55	217 ± 55	0.365
Blood biochemistry			
Retinol binding protein [ $M(P_{25},P_{75}),\mathrm{\mu g}/\mathrm{mL}$ ]	40.6(33.9,52.4)	42.2(33.2,48.8)	0.522
Parathyroid hormone [ $M(P_{25},P_{75}),\mathrm{pg}/\mathrm{mL}$ ]	48.5(40.2,59.8)	39.9(30.9,49.4)	<0.001
Serum uric acid [ $M(P_{25},P_{75}),\mu \mathrm{mol}/L$ ]	382.4(342.8,420.3)	376.9(286.9,439.9)	0.308
Urea [ $M(P_{25},P_{75}),\text{mmol}/L$ ]	5.5(4.6,6.7)	5.4(4.6,6.9)	0.742
Creatinine [ $M(P_{25},P_{75}),\mu \mathrm{mol}/L$ ]	76.6(65.5,90.0)	76.5(69.0,94.1)	0.401
eGFR [ $M(P_{25},P_{75}),\mathrm{ml}/\min/1.73m2$ ]	79.97(71.63,89.57)	81.78(71.11,95.90)	0.408
Total Cholesterol [ $M(P_{25},P_{75}),\text{mmol}/L$ ]	4.10(3.39,4.76)	4.15(3.27,4.74)	0.970
Triglyceride [ $M(P_{25},P_{75}),\text{mmol}/L$ ]	1.49(1.11,1.95)	1.47(1.02,2.14)	0.944
HDL-C [ $M(P_{25},P_{75}),\text{mmol}/L$ ]	1.07(0.92,1.25)	1.03(0.91,1.21)	0.516
LDL-C ( $\bar{x}\pm s,\text{mmol}/L$ )	2.53 ± 1.78	2.59 ± 1.93	0.642
Calcium [ $M(P_{25},P_{75}),\text{mmol}/L$ ]	2.28(2.21,2.35)	2.31(2.26,2.37)	0.020
Phosphorus [ $M(P_{25},P_{75}),\text{mmol}/L$ ]	1.19(1.10,1.31)	1.20(1.07,1.29)	0.743
Potassium [ $M(P_{25},P_{75}),\text{mmol}/L$ ]	3.82(3.57,4.06)	3.75(3.52,3.91)	0.091
Sodium [ $M(P_{25},P_{75}),\text{mmol}/L$ ]	140.7(139.3,142.2)	140.2(138.2,141.2)	0.004
Urinalysis			
Urinary potassium [ $M(P_{25},P_{75}),\text{mmol}/L$ ]	41(32,48)	43(35,56)	0.051
Urinary sodium [ $M(P_{25},P_{75}),\text{mmol}/L$ ]	156(113,179)	161(121,219)	0.086
Urinary chloride [ $M(P_{25},P_{75}),\text{mmol}/L$ ]	157(111,178)	161(125,214)	0.091
Uric acid [ $M(P_{25},P_{75}),\mu \mathrm{mol}/L$ ]	3.35(2.28,5.01)	4.04(2.96,5.57)	0.018
Urinary creatinine [ $M(P_{25},P_{75}),\mu \mathrm{mol}/L$ ]	10.3(7.7,12.8)	11.4(9.2,15.4)	0.004
Urinary microalbumin [ $M(P_{25},P_{75}),\mathrm{mg}/L$ ]	16(7,132)	16(7,88)	0.378
Total urinary protein [ $M(P_{25},P_{75}),\mathrm{mg}/L$ ]	112(70,249)	108(69,207)	0.327
Supine-to-standing test			
Orthostatic rennin [ $M(P_{25},P_{75}),\mathrm{uIU}/\mathrm{ml}$ ]	18.84(5.76,36.22)	16.18(8.37,34.94)	0.786
Orthostatic aldosterone [ $M(P_{25},P_{75}),\mathrm{ng}/\mathrm{dL}$ ]	13.91(9.21,20.20)	11.70(7.26,17.01)	0.018
Orthostatic ARR [ $M(P_{25},P_{75})$ ]	1.17(0.37,3.47)	0.80(0.32,1.90)	0.079
Supine renin [ $M(P_{25},P_{75}),\mathrm{uIU}/\mathrm{ml}$ ]	9.52(2.88,20.23)	7.68(3.54,16.56)	0.786
Supine aldosterone [ $M(P_{25},P_{75}),\mathrm{uIU}/\mathrm{ml}$ ]	9.42(6.49,14.40)	7.92(5.72,19.89)	0.006
Supine ARR [ $M(P_{25},P_{75})$ ]	1.63(0.55,4.80)	1.01(0.50,2.81)	0.049

### Binary logistic regression analysis of influencing factors of vitamin D deficiency

Logistic regression analysis was performed with vitamin D deficiency (25-OH-D < 50 nmol/L) as the dependent variable and the presence of primary aldosteronism, diabetes, hyperuricemia, coronary heart disease, hyperlipidemia, atrial fibrillation, smoking, and excessive alcohol consumption as independent variables.

Binary logistic regression analysis identified both excessive alcohol consumption (OR 1.331, 95%CI 0.624–2.838, *p* = 0.041) and primary aldosteronism (OR 0.210, 95%CI 0.045–0.974, *p* = 0.046) as factors independently associated with vitamin D deficiency (Table 4).

**Table 4:** Binary logistic regression analysis of influencing factors of vitamin D deficiency.

Variable	OR	95%CI	*P-*value
Primary aldosteronism	0.210	0.045–0.974	0.046
Diabetes	1.109	0.583–2.107	0.753
Hyperlipidemia	0.955	0.554–1.645	0.868
Hyperuricemia	0.657	0.334–1.293	0.224
Coronary heart disease	0.583	0.308–1.103	0.097
Atrial fibrillation	0.526	0.136–2.037	0.352
Smoking	1.752	0.916–3.351	0.090
Excessive alcohol consumption	1.331	0.624–2.838	0.041

### Multivariate linear regression analysis

The study revealed a significant relationship between decreased eGFR and low levels of 25-OH-D, even after accounting for factors like sex and serum potassium. The model was significant (*F* = 14.44, *p* < 0.001) and residuals were independent (Durbin–Watson = 2.01) (Tables 5, 6).

**Table 5:** Baseline characteristics of renal function and 25-OH-D.

25-OH-D (nmol/l)	CKD stage 1	CKD stage 2	CKD stage 3	CKD stage 4	CKD stage 5	Total
25-OH-D < 25	7	20	6	1	1	35
25 ≤ 25-OH-D < 50	31	83	14	1	1	130
50 ≤ 25-OH-D < 75	23	33	7	0	0	63
25-OH-D ≥ 75	5	15	2	0	0	22
Total	66	151	29	2	2	250

**Table 6:** Multivariable linear regression of estimated glomerular filtration rate (eGFR).

Variable	Model 1 (crude)	Model 2 (+demographics and lab)	Model 2 (+urinalysis)
25-OH-D(per 10 nmol/L)	0.050(0.455)	−2.543 (0.073)	−4.348(0.002)
Gender	–	−9.896(<0.001)	−11.845(<0.001)
Serum uric acid	–	−0.033(0.006)	−0.036(0.002)
Red blood cell count	–	3.350(0.095)	2.062(0.288)
Retinol binding protein	–	−0.572(<0.001)	−0.508(<0.001)
Parathyroid hormone	–	−0.094(0.003)	−0.098(0.001)
serum potassium	–	−8.753(<0.001)	−7.507(<0.001)
Ca × P	–	6.712(0.002)	6.524(0.001)
urinary potassium	–	–	0.112(0.106)
Uric acid	–	–	−0.023(0.603)
Urinary microalbumin	–	–	0.028(0.075)
Total urinary protein	–	–	−0.030(0.016)
Constant	78.143(<0.001)	140.208(<0.001)	145.187(<0.001)
Adj. *R*^2^	0.002	0.337	0.393

All coefficients represent the change in eGFR (ml/min/1.73 m^2^) per unit increase in the predictor. A statistically significant association was observed between serum 25-hydroxyvitamin D levels and estimated glomerular filtration rate (*β* = −4.348, *t* = −3.082, *p* = 0.002).

### The association between eGFR and cardiac structure and function

In the eGFR ≥60 mL/min/1.73 m^2^ group, the left atrial diameter[36(32,39) vs. 39(36,42), *p* = 0.001], pulmonary artery diameter[23(21,25) vs. 24(22,26), *p* = 0.010], and 24-h mean systolic blood pressure (127 ± 11 vs. 131 ± 11, *p* = 0.042) were all significantly lower, whereas 24-h mean diastolic blood pressure was significantly higher (77 ± 9 vs. 73 ± 10, *p* = 0.042), than in the eGFR <60 mL/min/1.73 m^2^ group (Table 7).

**Table 7:** Comparison of cardiac ultrasound and dynamic blood pressure data.

Characteristics	eGFR ≥ 60 ml/min/1.73m^2^ (*n* = 217)	eGFR < 60 ml/min/1.73m^2^ (*n* = 33)	*P-*value
Echocardiography			
AA [*M*(*P*_25_, *P*_75_), mm]	33(31,36)	34(32,38)	0.232
LVPW [*M*(*P*_25_, *P*_75_), mm]	10(9,11)	10(10,12)	0.258
LVDD [*M*(*P*_25_, *P*_75_), mm]	39(36,43)	40(39,44)	0.077
LAD [*M*(*P*_25_, *P*_75_), mm]	36(32,39)	39(36,42)	0.001
PAD [*M*(*P*_25_, *P*_75_), mm]	23(21,25)	24(22,26)	0.010
LVEDD [*M*(*P*_25_, *P*_75_), mm]	103(85,119)	106(86,120)	0.597
LVESD [*M*(*P*_25_, *P*_75_), mm]	40(34,48)	44(37,54)	0.113
IVSd [*M*(*P*_25_, *P*_75_), mm]	10(9,11)	11(9,12)	0.082
PV [*M*(*P*_25_, *P*_75_), cm/s]	95(86,104)	96(83,108)	0.813
LVW [*M*(*P*_25_, *P*_75_), mm]	45(42,48)	46(43,49)	0.283
LVAP [*M*(*P*_25_, *P*_75_), mm]	45(42,48)	45(41,48)	0.722
AAV [*M*(*P*_25_, *P*_75_), cm/s]	129(118,140)	130(123,139)	0.809
RAD [*M*(*P*_25_, *P*_75_), mm]	29(27,32)	30(27,32)	0.429
Blood pressure			
mSBP ( x±s,mmHg )	127 ± 11	131 ± 11	0.042
mDBP ( x±s,mmHg )	77 ± 9	73 ± 10	0.042
mHR [*M*(*P*_25_, *P*_75_), bpm]	71(66,75)	68(65,75)	0.378
MAP ( x±s,mmHg )	92 ± 9	92 ± 8	0.758
AASI ( x±s )	0.38 ± 0.19	0.46 ± 0.23	0.102

## Discussion

There is a growing body of research investigating the relationship between vitamin D and various diseases. Factors including geographic location, season, time of day, skin pigmentation, and sunscreen application can affect cutaneous vitamin D synthesis (4, 21, 22).

Emerging evidence has identified a potential association between PA and vitamin D deficiency. Specifically, individuals with PA have a higher prevalence of vitamin D deficiency, independent of confounding covariates (8, 9). In multiple linear regression analysis, vitamin D deficiency was independently associated with PA occurrence (OR 0.210, 95%CI 0.045–0.974, *p* = 0.046) (see Table 3). In the supine position test, participants with serum 25-OH-D < 50 nmol/L had significantly higher supine aldosterone concentrations [9.42(6.49,14.40) vs. 7.92(5.72,19.89), *p* = 0.006], supine ARR [1.63(0.55,4.80) vs. 1.01(0.50,2.81), *p* = 0.049], and orthostatic aldosterone concentrations [13.91(9.21,20.20) vs. 11.70(7.26,17.01), *p* = 0.018] compared with those with serum 25-OH-D ≥ 50 nmol/L, with statistically significant between-group differences (see Table 2). Although the between-group difference in ARR reached statistical significance only in the supine position, this finding is likely attributable to the strong confounding effects of upright posture.

This suggests that vitamin D deficiency may be associated with the pathophysiological progression of PA. The underlying mechanism is thought to involve vitamin D-mediated regulation of central mineralocorticoid receptors (MR) (8), in conjunction with direct suppression of aldosterone synthase by 1α,25-(OH)_2_-D (10).

Aldosterone, a lipophilic mineralocorticoid, readily crosses the blood–brain barrier and binds to central mineralocorticoid receptors (MR), leading to increased sympathetic activity and subsequent renal vasodilation (23, 24). The resultant sympathetic drive induces renal vasodilation, diminishes proximal tubular reabsorption, and augments distal tubular delivery of Na^+^ and Ca^2+^.

Within renal proximal-tubular epithelial cells, 25-OH-D is 1α-hydroxylated by mitochondrial CYP27B1 to produce the biologically active metabolite 1,25-(OH)₂-D. Vitamin D and parathyroid hormone (PTH) are intricately involved in the maintenance of calcium homeostasis, with PTH stimulating the conversion of vitamin D to its biologically active form, 1,25-(OH)₂-D (25–27). Vitamin D and PTH exhibit reciprocal regulation: PTH binds to receptors on the basolateral membrane of renal proximal tubules, upregulates CYP27B1 transcription, and drives the conversion of 25-OH-D to 1,25-(OH)_2_-D. This accelerated 1α-hydroxylation consequently increases vitamin D catabolism and reduces circulating 25-OH-D levels (5, 28–31). Previous studies have shown that vitamin D supplementation can reduce plasma aldosterone concentrations in patients with hypertension, indicating vitamin D as a potential therapeutic target for patients with primary aldosteronism (PA) (9, 32). *In vitro* experiments have demonstrated that biologically active vitamin D can directly inhibit aldosterone synthesis, further corroborating the link between vitamin D deficiency and PA. Additionally, oxidative stress and chronic inflammation associated with PA can impair the synthesis of biologically active vitamin D in the liver and kidneys, thereby exacerbating vitamin D deficiency. Vitamin D receptor (VDR) is widely expressed in human tissues, with the highest expression levels detected in immune and cardiovascular cells. In PA, downregulated VDR expression attenuates vitamin D-mediated negative feedback regulation. Consequently, a state of functional vitamin D insufficiency may develop even in the presence of normal serum 25-OH-D concentrations. Further investigations are warranted to elucidate the precise underlying mechanisms and to explore potential vitamin D pathway-targeted therapeutic interventions for the management of PA (33).

Vitamin D deficiency is widely recognized as a cardinal clinical feature of chronic kidney disease-mineral and bone disorder (CKD-MBD). However, the relationship between impaired renal function and reduced vitamin D status is bidirectional: vitamin D deficiency itself can reduce glomerular filtration rate (GFR) through multiple pathophysiological mechanisms. Low serum 25-OH-D levels have been associated with increased renin-angiotensin-aldosterone system (RAAS) activity (32). The RAAS is a major hemodynamic regulatory system that plays a well-established pivotal role in blood pressure regulation and sodium-water homeostasis. Experimental evidence has shown that inhibition of 1,25-(OH)₂-D synthesis leads to increased renin expression. Conversely, exogenous administration of 1,25-(OH)₂-D results in the inhibition of renin activity (33). Binding of 1,25-(OH)₂-D to VDR directly represses renin gene transcription, most likely by disrupting the assembly of cyclic AMP response element-binding protein (CREB) complexes at the renin gene promoter. Furthermore, vitamin D deficiency can stimulate renin secretion, leading to RAAS activation. This, in turn, increases angiotensin II production, which further promotes aldosterone synthesis and secretion by zona glomerulosa cells of the adrenal cortex. Clinical studies have indicated that vitamin D supplementation can lower blood pressure in patients with essential hypertension. Notably, exogenous 1,25-(OH)₂-D administration not only reduces blood pressure but also decreases plasma renin activity and circulating angiotensin II levels (33). Angiotensin II, a potent bioactive vasoconstrictor, induces vascular smooth muscle contraction, thereby causing generalized vasoconstriction, including that of efferent arterioles. This elevates intraglomerular pressure, impairs the glomerular filtration barrier, and ultimately results in reduced GFR. Angiotensin II is also subject to a negative feedback loop: under normal physiological conditions, expansion of the circulating blood volume suppresses renin secretion, thereby reducing angiotensin II concentrations and blood pressure. In contrast, aldosterone disrupts this normal negative feedback loop, and stimulates the release of transforming growth factor-β (TGF-β) from distal tubular epithelial cells and interstitial fibroblasts. This subsequently activates NADPH oxidase and increases reactive oxygen species (ROS) generation. ROS sensitize the Na^+^-K^+^-2Cl^−^ cotransporter (NKCC2) on the luminal membrane of distal tubules, impair macula densa signaling, and thereby sustain juxtaglomerular renin secretion. This leads to persistent elevation of angiotensin II levels, which perpetuates high systemic vascular resistance and intraglomerular pressure. These pathological changes may eventually lead to glomerulosclerosis, tubulointerstitial fibrosis, and progressive decline in estimated GFR (eGFR). Vitamin D deficiency stimulates a compensatory increase in circulating fibroblast growth factor 23 (FGF-23) to maintain phosphate homeostasis. Elevated serum phosphate and FGF-23 levels suppress renal tubular 1α-hydroxylase activity, further reducing 1,25-(OH)₂-D synthesis, exacerbating renal injury, and contributing to progressive GFR decline. Additionally, vitamin D modulates multiple signaling pathways and exerts anti-inflammatory and anti-fibrotic effects. It enhances mitogen-activated protein kinase (MAPK) activity, inhibits nuclear factor-κB (NF-κB) signaling, regulates tumor-immune cell crosstalk to control cytokine production, and influences prostaglandin metabolism (34, 35). Vitamin D deficiency decreases IκB*α* expression, thereby amplifying NF-κB activity; this in turn drives the release of pro-inflammatory cytokines including IL-6 and TNF-α, induces tubulointerstitial inflammation and fibrosis, and ultimately impairs glomerular filtration rate (GFR).

Patients with chronic kidney disease (CKD) have a significantly elevated risk of cardiovascular disease (CVD) compared with individuals without CKD (36). The renin-angiotensin-aldosterone system (RAAS) plays a critical role in maintaining blood pressure and fluid volume homeostasis, and is additionally implicated in the pathogenesis of cardiovascular dysfunction (37, 38). Emerging evidence suggests that vitamin D exerts negative regulatory effects on the RAAS, which is likely the primary mechanistic link between vitamin D status and hypertension (39). Although no direct association was identified between vitamin D deficiency and structural or functional cardiac abnormalities, participants with CKD exhibited a significantly larger left atrial anteroposterior diameter and pulmonary artery diameter, alongside higher 24-h mean systolic blood pressure (SBP) but lower 24-h mean diastolic blood pressure (DBP), compared with those without renal dysfunction. No statistically significant between-group difference was observed in arterial stiffness index. These findings likely reflect increased volume overload and suboptimal blood pressure control in patients with CKD, whereas the reduced diastolic blood pressure in this population may indicate advanced arterial stiffening. These observations further indicate that vitamin D deficiency may alter cardiac morphology via RAAS-dependent pathways, and suggest that vitamin D supplementation may mitigate hypertension and renal injury risk, but further experiments are needed to confirm this (39).

### Limitations

In our research, residual confounding by seasonal variation, sunlight exposure and physical activity was not adjusted for, and this could have affected our findings. However, given the cross-sectional design, consecutive enrolment and the fact that all participants were hospitalized inpatients, we believe that season, ambient sunlight and habitual physical activity are unlikely to have introduced critical confounding bias. Our study was underpowered for definitive assessments of echocardiographic parameters; therefore, the observed cardiac-related results are strictly preliminary and hypothesis-generating only. The cross-sectional nature of our study inherently limits our ability to infer the direction or temporality of the observed associations, as exposure and outcomes were assessed concurrently. Consequently, our findings reflect only statistical correlations rather than evidence of causality, and no causal or predictive inferences can be established from the present data. Prospective interventional studies are warranted to clarify whether vitamin D supplementation may reduce the incidence of PA and ameliorate related cardiovascular and renal dysfunction.

## Conclusion

After adjustment for conventional cardiovascular risk factors, primary aldosteronism (PA) remained independently associated with vitamin D deficiency (OR 0.210, 95%CI 0.045–0.974, *p* = 0.046). Vitamin D and retinol-binding protein (RBP) were significantly associated with renal dysfunction in the study population. Vitamin D deficiency might indirectly affect cardiac structure and function through renal-mediated pathways; these findings are preliminary and strictly hypothesis-generating due to limited statistical power, and further experimental validation is required. These cross-sectional data demonstrate only statistical associations; causality cannot be inferred. We advocate for systematic screening and correction of vitamin D deficiency in patients with primary aldosteronism; however, current evidence does not support the use of supraphysiological vitamin D dosing to confer additional cardiovascular or renal protective benefits.

## Data Availability

The raw data supporting the conclusions of this article will be made available by the authors, without undue reservation.
